# Comparison of Various Functionally Graded Femoral Prostheses by Finite Element Analysis

**DOI:** 10.1155/2014/807621

**Published:** 2014-08-27

**Authors:** Azim Ataollahi Oshkour, Hossein Talebi, Seyed Farid Seyed Shirazi, Mehdi Bayat, Yat Huang Yau, Faris Tarlochan, Noor Azuan Abu Osman

**Affiliations:** ^1^Department of Mechanical Engineering, Faculty of Engineering, University of Malaya, 50603, Kuala Lumpur, Malaysia; ^2^Research Training Group GRK 1462, Bauhaus-Universität Weimar, Berkaer Straße 9, 99425 Weimar, Germany; ^3^Department of Civil Engineering, Aalborg University, Sohngårdsholmsvej 57, 9000 Aalborg, Denmark; ^4^Department of Mechanical and Industrial Engineering, College of Engineering, Qatar University, Doha 2713, Qatar; ^5^Department of Biomedical Engineering, Faculty of Engineering, University of Malaya, 50603 Kuala Lumpur, Malaysia

## Abstract

This study is focused on finite element analysis of a model comprising femur into which a femoral component of a total hip replacement was implanted. The considered prosthesis is fabricated from a functionally graded material (FGM) comprising a layer of a titanium alloy bonded to a layer of hydroxyapatite. The elastic modulus of the FGM was adjusted in the radial, longitudinal, and longitudinal-radial directions by altering the volume fraction gradient exponent. Four cases were studied, involving two different methods of anchoring the prosthesis to the spongy bone and two cases of applied loading. The results revealed that the FG prostheses provoked more SED to the bone. The FG prostheses carried less stress, while more stress was induced to the bone and cement. Meanwhile, less shear interface stress was stimulated to the prosthesis-bone interface in the noncemented FG prostheses. The cement-bone interface carried more stress compared to the prosthesis-cement interface. Stair climbing induced more harmful effects to the implanted femur components compared to the normal walking by causing more stress. Therefore, stress shielding, developed stresses, and interface stresses in the THR components could be adjusted through the controlling stiffness of the FG prosthesis by managing volume fraction gradient exponent.

## 1. Introduction

Total hip replacement (THR) is regarded as a last resort but it is a very effective procedure to relieve pain and restore the function of a degenerated hip joint [1]. Insertion of a prosthesis into the femur alters the bone stress pattern because of the mismatch between the stiffness of the prosthesis and that of the existing bone [2, 3]. Given their stiffness relative to that of bones, prostheses shield against stress transformation from the hip joint to the proximal part of the femur [2, 4]. The bone positioned along the engineered materials is a live tissue and can thus adapt itself to the new mechanical and chemical environment. With stress shielding of the prosthesis, the cortical structure of the bone loses its strength [2, 5]. Stress shielding leads to aseptic loosening, the leading cause of failure of THRs [4, 6, 7]. Efforts have been directed towards identifying appropriate materials for fabricating prostheses so that stress shielding may be minimized. Consequently, composite materials have been used in femoral prostheses [8–12]. Among the composite materials available, FGMs have drawn special interest because they exhibit interesting properties that have the potential to minimize stress shielding.

FGM displays a continuous (gradient) or a stepwise (graded) change in its microstructure and, hence, properties. The concept of FGMs is based on natural biological structures [13]. The mechanical and structural properties of a FGM may be controlled and, hence, optimized by adjusting the volume fraction of each of its constituent phases [14]. Compared with their monolithic ceramic or metallic counterparts, FGMs have higher load-bearing, fracture toughness, wearing resistance [15–17], and biocompatibility [18–20]. As such, an FGM is an attractive candidate for fabricating prostheses, in particular joint fixation devices, such as the femoral component of a THR [14, 21]. The use of FGMs in orthopedic prostheses may be traced to their use in dental implants. Examples include the study of bone remodeling induced by dental implants [22], design optimization of dental implant for bone remodeling [23], and thermomechanical study of dental implants [24].

Kuiper and Huiskes [25] developed a numerical design optimization method and coupled it with 2D finite element analysis (FEA) to find a solution for the problem of decreasing stress shielding without inducing excessive interface stress. They found that a prosthesis with tailorable stiffness could help to limit bone loss and interface stresses. Their model was limited to the 2D finite element analysis and a single loading case of a pure bending moment. Simões et al. [26] followed findings of Kuiper and Huiskes [25] and they developed a composite prosthesis and a metal core with a variable stiffness. They controlled the stiffness of prosthesis by adjusting the thickness of the composite layer around the metal core and they achieved more SED and minimum principal stress in the bone. They performed a simplified 2D FEA and applied a vertical load of 3 kN on the femoral prosthesis. Hedia et al. [27, 28] made a 2D model of prostheses composed of FGMs and different gradient directions and accessed their performance by a 2D FEA. They showed more stress in the bone and reduction in interface stress owing to the use of the FGMs in the femoral prosthesis. However similar to their previous studies, this was limited to the 2D with a single load case. Moreover, they exploited ceramic materials with low fracture toughness. A numerical study was performed by Gong et al. [29] on the adaptation of bone due to impact of materials of the noncemented femoral stem. They found that the FG prostheses preserved the host bone better than the prostheses with conventional materials owing to having more mechanical stimuli, more uniform interface shear stress, and smaller maximum interface stress. A three-dimensional FEA was conducted by Oshkour et al. [30] to determine the performance of cemented FG prostheses with a longitudinal gradient direction during a gait. They found less stress in the FG prostheses and more stress develops in the bone and the cement. The cemented prostheses with longitudinal change in the modulus of elasticity were only considered in this work.

All these research studies just mentioned have their own merits; however they are mainly limited to 2D FEA simulation on noncemented prostheses with simplified models of loadings cases. Therefore, there is limited information about the impact of FGMs on the developed stress in the implanted femur components to assess the risk of failure. Moreover, the previous studies employing 3D models were limited to the change in the modulus of elasticity of prosthesis in longitudinal direction without presenting the SED and interface stresses. In addition, there is no study on the performance of FGMs during the stair climbing in which it will induce more detrimental torsional load and interface stress on the implanted femur constituents [31]. Therefore, in the present study the FEA was used to analyze a model of a femur implanted with a FG prosthesis (femoral component of a THR), subjecting to loading experienced during normal walking (maximum contact force) or stair climbing (maximum torsional moment). For each loading condition, two cases were considered: implant cemented in the femur (cemented case) or pressfitted into the bone (noncemented cases). For each combination of implant fixation method and applied loading, the following parameters were determined: strain energy density and developed stress in prostheses, bone, and cement and stress at the implant-bone interface.

## 2. Materials and Methods

### 2.1. Modeling and Meshing

**Figure 1:**
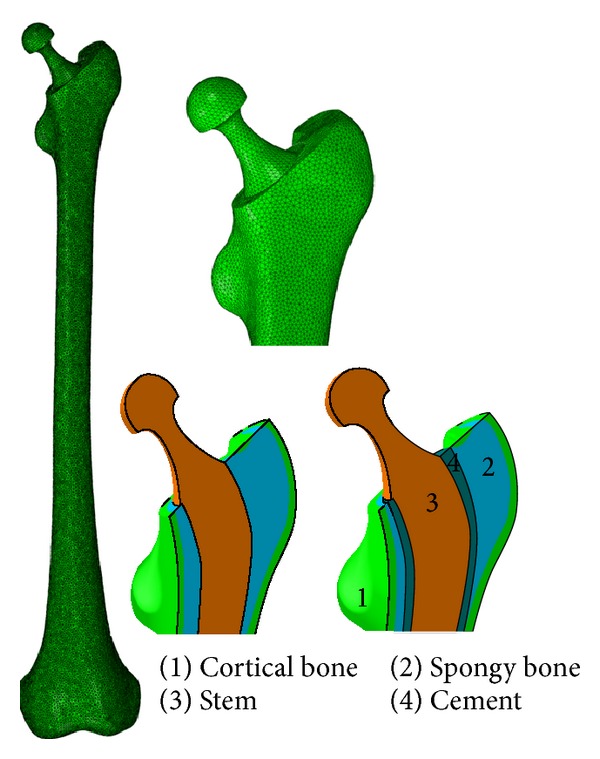
The implanted femur model.

A three-dimensional (3D) model of a human femur was developed based on computed tomography images of the bone. A total of 998 images with 512 pixels × 512 pixels and a spatial resolution of 0.549 mm were captured using a multidetector Siemens unit (Sensation 64; Siemens Medical Solutions, Malvern, PA, USA). The images were exported to the Mimics software (version 13; Materialize NV, Leuven, Belgium) to extract a 3D model of the femur. The Charnley femoral prosthesis and the cement layer were developed using Pro/Engineer software (version 5; Parametric Technology Corporation, Needham, MA, USA). Ebramzadeh et al. [36] reported that the optimum thickness for the cement layer ranges from 2 mm to 5 mm. Joshi et al. [37] also indicated reduced incidence of osteolysis when a cement layer thickness of 3 mm is employed, thus, in the present study, a cement layer thickness of 3 mm was used. Models of the bone, the cement layer, and the prosthesis were exported to the finite element software package (ABAQUS Inc., Providence, RI, USA), where they were assembled into a single finite element (FE) model (Figure 1) and then meshed using 3D tetrahedral elements [38] (Table 1). A convergence test was performed on the FE model.

### 2.2. Boundary Conditions

**Figure 2:**
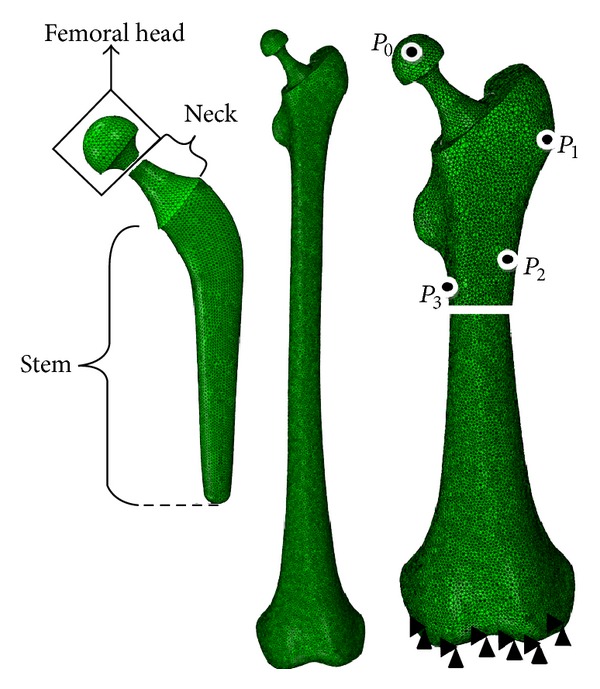
Mesh and boundary condition.

Static analysis was employed to simulate simplified loading configurations on the implanted femur, with the highest contact force and the highest torsional moment in normal walking and in stair climbing, respectively. The forces during normal walking and stair climbing are shown in Tables 2 and 3, respectively. The hip contact force and muscle loading at the hip joint were based on a study by Bergmann et al. [32] and Heller et al. [33]. The locations of the applied forces are shown in Figure 2. The femur was fixed at the distal end of the knee joint (Figure 1) [30, 39]. The bone and the cement layer were bonded in the cemented prosthesis implantation [40]. Surface-to-surface contact with finite sliding and a friction coefficient of 0.3 were considered for bone prosthesis in the noncemented implantation [34, 41] and the prosthesis-cement interface in the cemented implantation [35, 42]. To apply the loads and the material property to the cortical bone, a datum coordinate system was defined in ABAQUS, with the *z*-axis parallel to the idealized midline of the femur and the *x*-axis parallel to the dorsal contour of the femoral condyles in the transverse plane [32].

### 2.3. Materials

**Figure 3:**
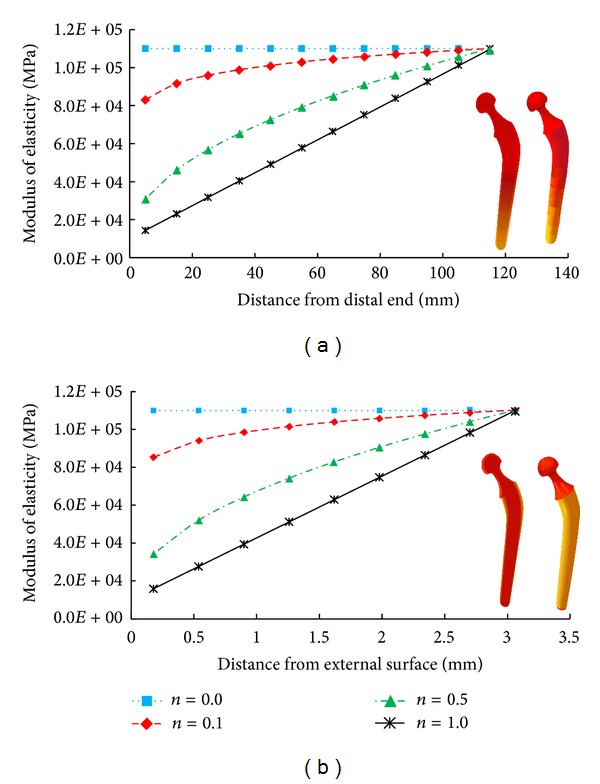
Variation of modulus of elasticity (a) in longitudinal direction from distal to proximal when the radial volume fraction gradient is 0 and (b) in radial direction from external surface to internal core when the longitudinal volume fraction gradient is 0.

The mechanical properties of the materials are presented in Table 4 [30, 35, 42]. The cortical bone was considered a transversely isotropic elastic material, whereas the spongy bone, cement, hydroxyapatite (HA), and titanium alloy (Ti) were considered linear isotropic elastic materials. To assign material properties of the cortical bone, elastic properties were inserted into ABAQUS by selecting the type of engineering constants. The datum coordinate system was subsequently employed to orient the properties. The FG prosthesis comprised layers of Ti and HA, with the variation of the modulus of elasticity of the FGM (*E*), along the longitudinal and radial directions, being described by the following power law [43]:

(1) $$\begin{array}{c} E=E_{\text{Ti}}{\left(\frac{2K+h}{2h}\right)}^{b}+E_{\text{HA}}\left(1-{\left(\frac{2K+h}{2h}\right)}^{b}\right),\\ b=\text{volume}\;\text{fraction}\;\text{gradient}\;\text{exponent},\\ b=m\left(\text{longitudinal}\;\text{volume}\;\text{fraction}\;\text{gradient}\;\text{exponent}\right),\\ b=n\left(\text{radial}\;\text{volume}\;\text{fraction}\;\text{gradient}\;\text{exponent}\right),\\ 0\leq n\leq \infty ,\\ 0\leq m\leq \infty ,\\ -\frac{h}{2}\leq K\leq \frac{h}{2}, \end{array}$$

where *E*_Ti_ and *E*_HA_ are the modulus of elasticity for Ti and HA, respectively; *h* represents the height (115.0 mm) and the thickness (3.1 mm) of the prosthesis in the longitudinal and longitudinal and radial directions, respectively; *K* denotes the change in stem height in the longitudinal direction of the prosthesis from the distal to the proximal end and the change in thickness in the radial direction of the prosthesis from the cortex layer to the central core; and *n* and *m* are the radial and the longitudinal volume fraction gradient exponents, respectively. The volume fraction gradient exponents of 0.0, 0.1, 0.5, and 1.0 were employed to alter the value of *E* in the radial and the longitudinal directions; the volume fraction gradient exponent of 0.0 represented Ti. The variation of *E* with *n* and *m* in the longitudinal and radial directions of the prosthesis is presented in Figure 3.

## 3. Results

**Figure 4:**
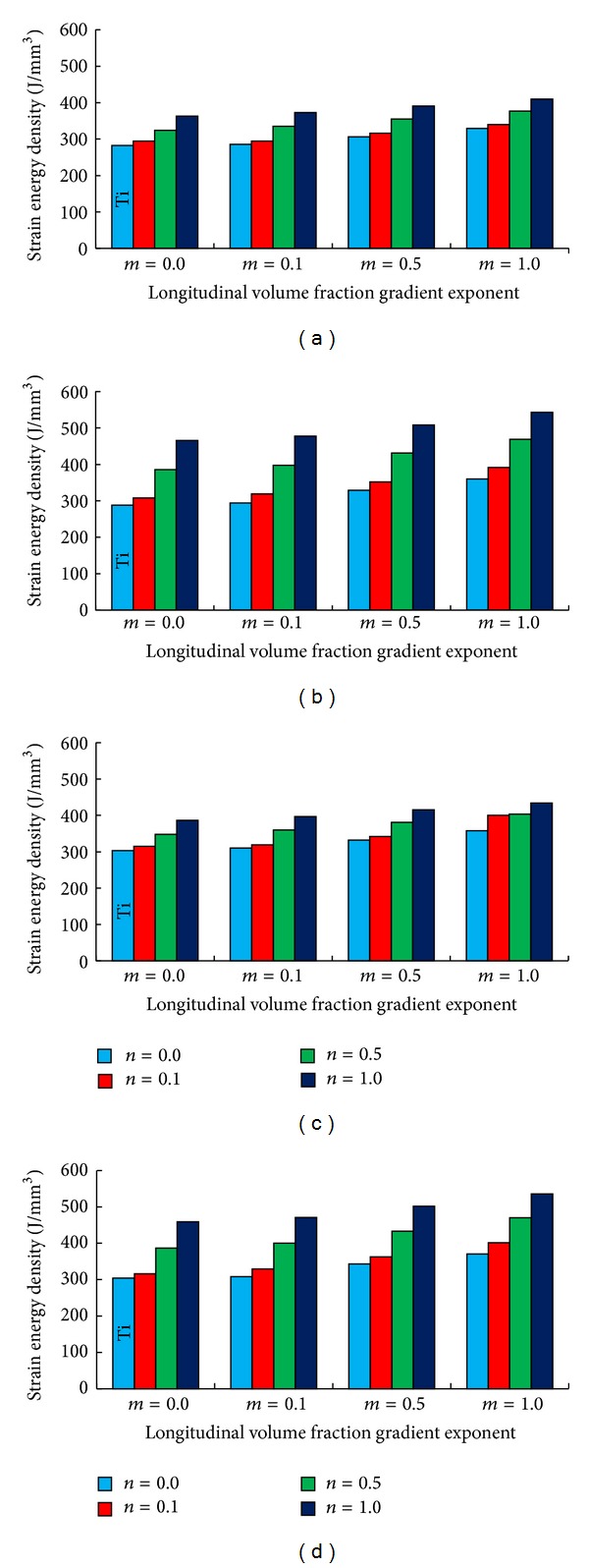
Strain energy in the spongy portion of the proximal metaphysis of the femur due to the implantation of (a) normal walking-cemented prostheses, (b) normal walking-non-cemented prostheses, (c) stair climbing-cemented prostheses, and (d) stair climbing-non-cemented prostheses (the legend shows the radial volume fraction gradient exponent change). *n* is radial volume fraction gradient exponent.

The variation of the strain energy density (SED) in the spongy portion of the proximal metaphysis of the femur after implantation with the different FG femoral prostheses is shown in Figure 4. It is seen that (1) the lowest SED was obtained with *n* = 0.0 and *m* = 0.0 (Ti, conventional material), whereas the highest SED was obtained with *n* = 1.0 and *m* = 1.0; (2) an increase in *n* produced about two times the effect on SED as an increase in *m*; (3) SED for a noncemented FG prosthesis was higher than that for a cemented one; and (4) SED was lower under normal walking conditions than under stair climbing.

**Figure 5:**
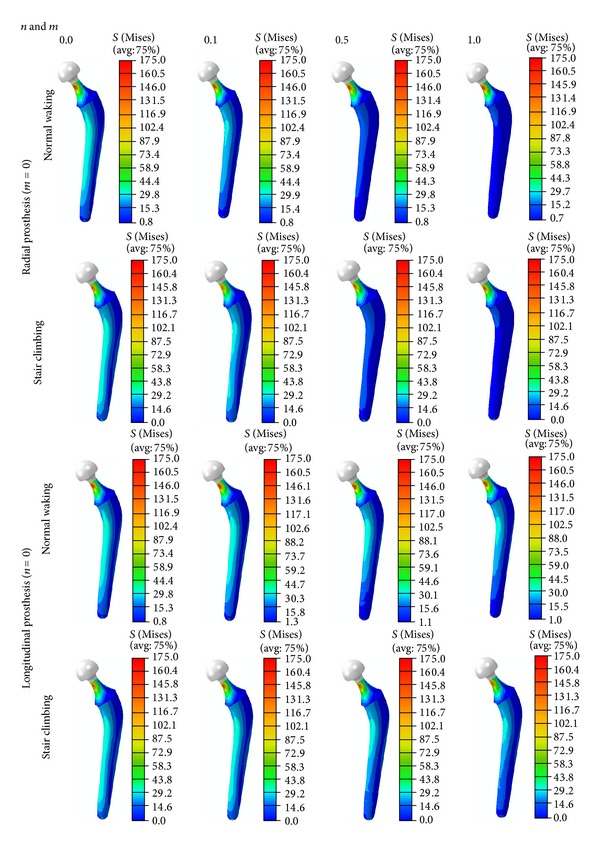
The von Mises stress distribution in the radial and longitudinal directions of the noncemented prostheses, under normal walking and stair climbing conditions.

**Figure 6:**
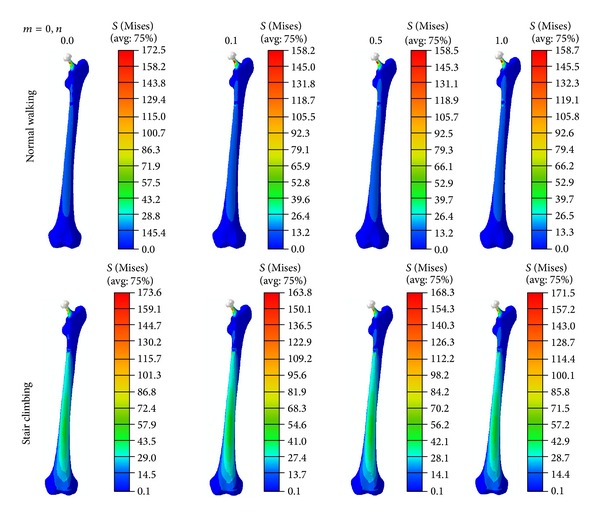
The von Mises stress distribution in the femur due to inserting radial noncemented prosthesis, under normal walking and stair climbing conditions.

During either normal walking or stair climbing, (1) the von Mises stress in the noncemented FG prosthesis decreased in both the radial and the longitudinal directions of the prosthesis with increase in both *n* and *m* (Figure 5) and (2) the von Mises stress in the femur, when a noncemented FG prosthesis was implanted, was significantly affected (Figure 6).

**Figure 7:**
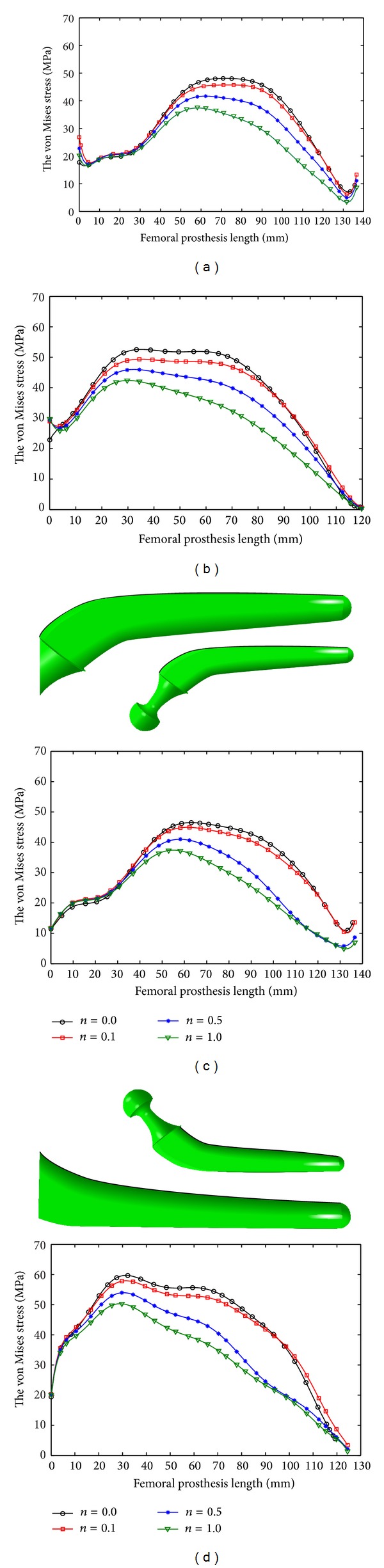
The von Mises stress variation on the longitudinal femoral prosthesis under normal walking: (a) lateral side of cemented prosthesis, (b) medial side of cemented prosthesis, (c) lateral side of noncemented prosthesis, and (d) medial side of noncemented prosthesis.

**Figure 8:**
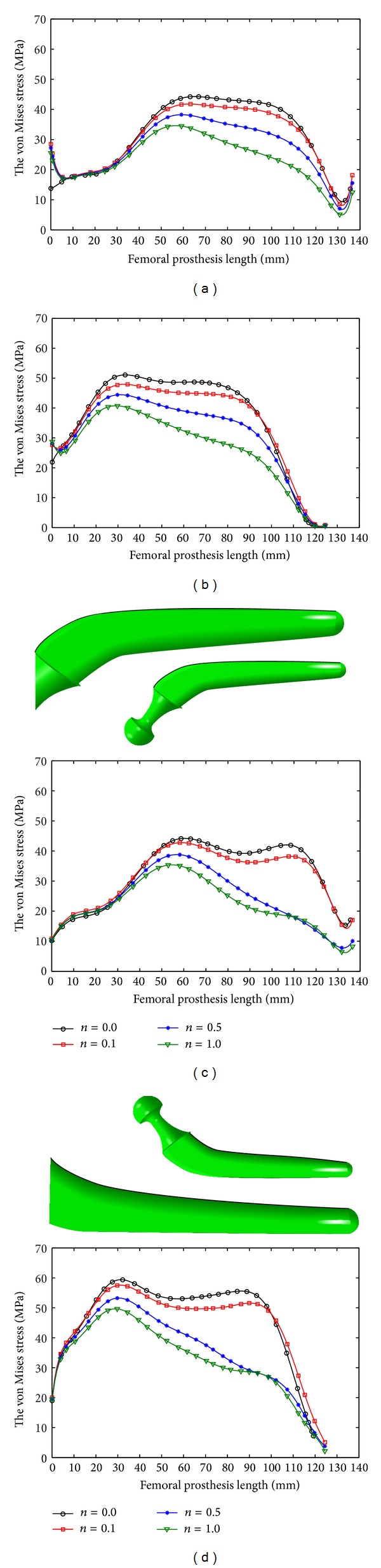
The von Mises stress variation on the longitudinal femoral prosthesis under stair climbing: (a) lateral side of cemented prosthesis, (b) medial side of cemented prosthesis, (c) lateral side of cementless prosthesis, and (d) medial side of cementless prosthesis.

The peak value of the von Mises stress in the prosthesis decreased with increase in *n* and *m* (Figures 7 and 8; Table 5) and the noncemented prostheses experienced lower stress than the cemented ones (Figures 7 and 8; Table 5), and more stress was exerted to the medial side of prostheses compared to the lateral side (Figures 7 and 8; Table 5).

**Figure 9:**
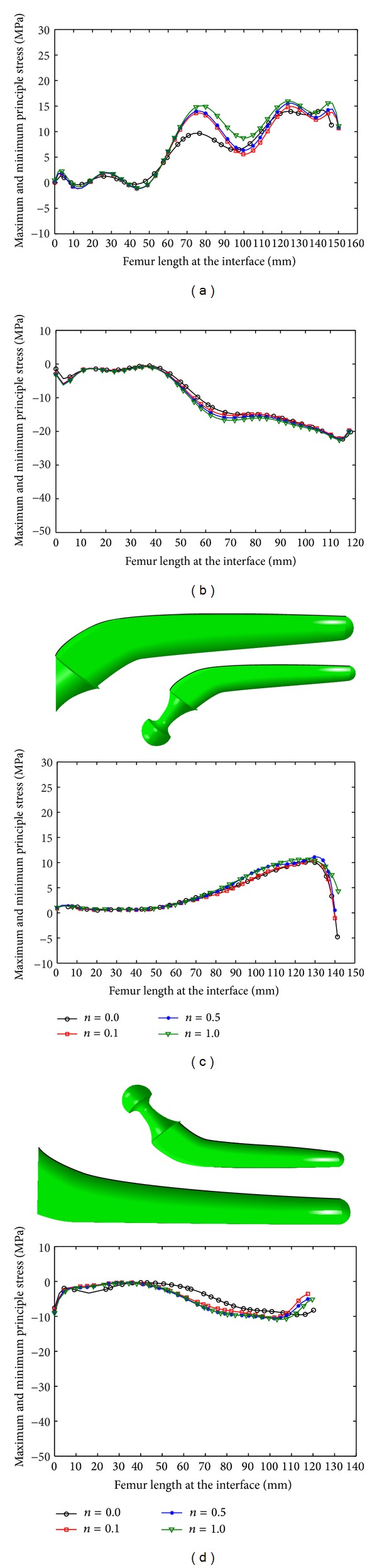
Stress variation on the internal surface of the femur under normal walking: (a) maximum principal stress, cemented prosthesis, (b) minimum principal stress, cemented prosthesis, (c) maximum principal stress, noncemented prosthesis, and (d) minimum principal stress, noncemented prosthesis.

**Figure 10:**
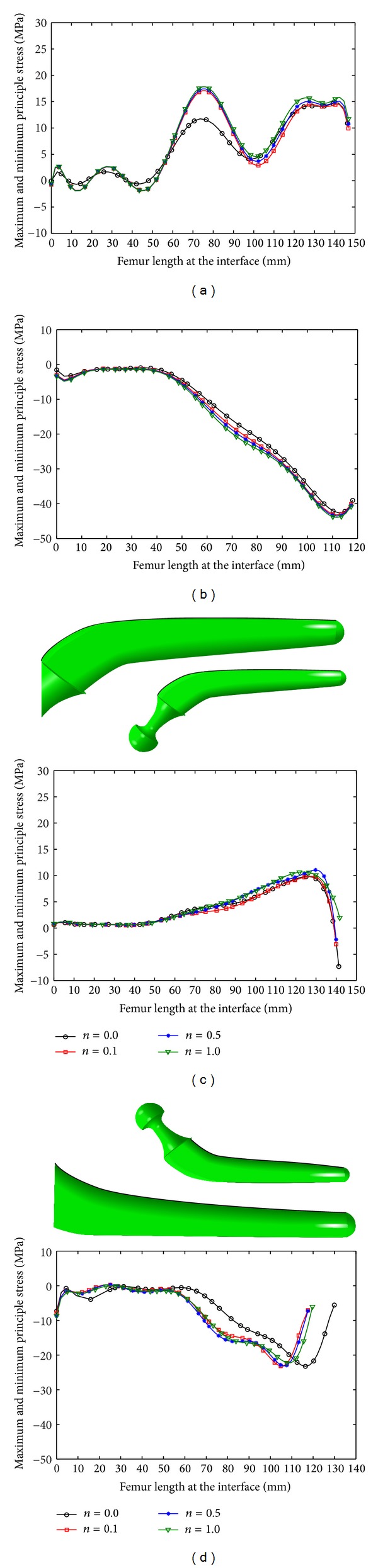
Stress variation on the internal surface of the femur under stair climbing: (a) maximum principal stress, cemented prosthesis, (b) minimum principal stress, cemented prosthesis, (c) maximum principal stress, noncemented prosthesis, and (d) minimum principal stress, noncemented prosthesis.

The peak value of the maximum and minimum principal stresses in the femur increased with increase in *n* and *m* (Figures 9 and 10; Table 6); insertion of the noncemented prosthesis produced lower stress than when the cemented one was inserted (Figures 9 and 10; Table 6), more stress was induced in the bone under the stair climbing than the normal walking condition (Figures 9 and 10; Table 6) and medial side of the bone carried more stress in comparison with the lateral side (Figures 9 and 10; Table 6).

**Figure 11:**
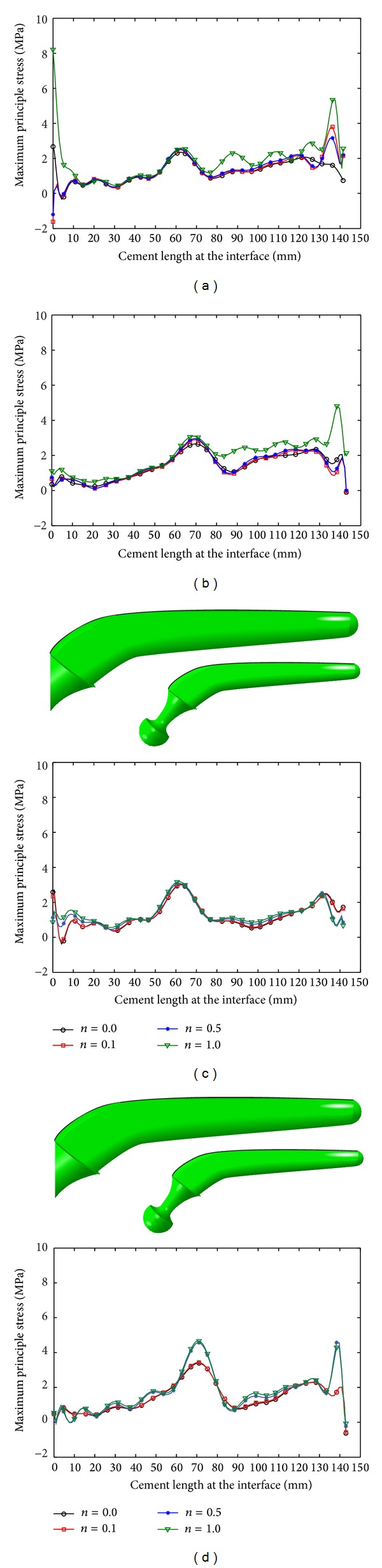
Maximum principal stress variation on the cement surface due to longitudinal prosthesis: (a) normal walking, internal surface, (b) normal walking, external surface, (c) stair climbing, internal surface, and (d) stair climbing, external surface.

**Figure 12:**
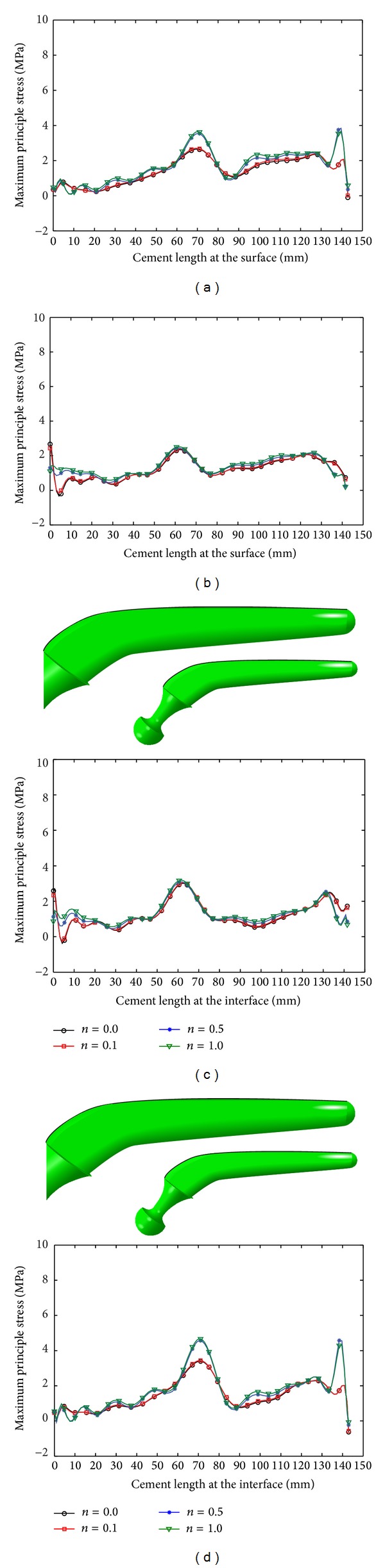
Maximum principal stress variation in cement layer due to radial prosthesis: (a) normal walking, internal surface, (b) normal walking, external surface, (c) stair climbing, internal surface, and (d) stair climbing, external surface.

The peak value of the maximum principal stress on the surface of the cement layer increased with increase in *n* and *m* (Figures 11 and 12; Table 7) and low stress was obtained at the external surface of the cement layer, when normal walking condition was used, whereas high stress was obtained at the external surface of the cement layer, when stair climbing condition was used (Figures 11 and 12; Table 7).

In the cemented model, (1) at both the prosthesis-cement interface and the cement-bone interface, the stress was practically constant with increase in *m* and *n* (Table 8) and (2) the stress at the cement-bone interface was higher than that at the prosthesis-cement interface (Table 8). In the noncemented model, at both the prosthesis-cement interface and the cement-bone interface, (1) the stress decreased with increase in *m* and *n* (Table 8) and (2) the stress under stair climbing condition was higher than that under normal walking condition (Table 8).

## 4. Discussion

The limited lifespan of the THR is highly considered by the surgeons and prosthetists due to the complications of the revision surgeries [30]. Therefore, they make an effort to increase the longevity of the THR by improving surgery methods and designs. Stiffness of the prosthesis by affecting the stress shielding and interface stresses plays a significant role in the durability of the THR. Prosthesis stiffness is a function of the prosthesis material and cross-section geometry [4]. Therefore, to minimize stress shielding and interface stresses prosthetist tries to optimize prosthesis stiffness by employing new materials in the prosthesis design. As a result, in many studies researchers have employed FEA in conjunction with the FGMs and made an effort to introduce a new design that could find an optimum compromise between stress shielding and interface stresses [27–29]. However, their works were limited to the 2D FEA, simplified models of the noncemented prostheses and subjected to a single simplified load case. Therefore, in the present work, a 3D FEA was exploited to examine performance of the noncemented and cemented FG prostheses and explore more about the stress distribution in the THR constituents (femoral stem, bone, and cement) in the two loading cases of the highest contact force in the normal waking and the highest torsional moment in the stair climbing.

Volume fraction of phases in the FGMs is adjusted by the volume fraction gradient exponent (*n* and *m*). In the present study, the volume fraction of ceramic phase with less modulus of elasticity increased by the volume fraction gradient exponent growth. Since, the stiffness of the prosthesis is a function of the modulus of elasticity of the prosthesis, the stiffness of prostheses declines by volume fraction gradient exponent growth. Moreover, it has been shown that the induced SED in the proximal metaphysis of the femur has an adverse relationship with the stiffness of prosthesis. Therefore, more SED was stimulated to the proximal portion of the femur by FG prostheses compared to the prostheses made of conventional material of Ti (*n* = 0 and *m* = 0) by increase in the *n* and *m*. The increase in the *n* and *m* simultaneously provoked more SED in the bone compared to the individual *n* and *m* growth due to more reduction in the prosthesis stiffness. Meanwhile, the radial volume fraction gradient exponent (*n*) showed more contribution in the SED enhancement in comparison with the longitudinal volume fraction gradient exponent (*m*). In the cemented prosthesis implantation, a portion of the loads is carried and damped by the cement layer. Therefore, the volume fraction gradient exponent growth was less influential on the SED increase in the cemented fixation method than the noncemented prosthesis implantation.

Loads transfer mechanism at the proximal of the femur alters after the THR. In other words, loads transfer to the femur though the femoral stem from the hip joint and the loads are partially transferred through shear across the bone, cement, and prostheses interfaces [44]. The stiffness of the THR components (prosthesis, cement, and bone) plays a significant role and dictates amounts of the load sharing between them. The prostheses composed of the conventional materials (Ti, chrome-cobalt, and stainless steel) are stiffer than the cortical bone [45]. Therefore, more loads are carried by prostheses compared to the bone and cement at the proximal portion of the femur. However, in the THR with FG prostheses, the prostheses share more loads with the bone and cement at the proximal portion of the femur with the volume fraction gradient exponent growth. This is due to the decrease in the mismatching between the stiffness of the prosthesis with the bone and cement as a result of the stiffness reduction of the prosthesis. Therefore, less stress was induced to the FG femoral stem than the femoral stem composed of Ti (*n* = 0 and *m* = 0), while the bone and cement tolerate more stress. However, the stress increase in the bone and cement is much less than the ultimate tensile strength of bone (121 MPa), the ultimate compressive strength of the bone (167 MPa), and the ultimate tensile strength of the cement (30 MPa). Meanwhile, in the FG prostheses, the stress distribution pattern on the prosthesis is altered by increase in the volume fraction gradient exponent and the peak value of the stresses declines on the surface of the prosthesis. Therefore, the FG prostheses provoke less interface stress even with reduction in the stiffness of the prosthesis especially for noncemented prostheses.

The FG prostheses induced more SED to the proximal metaphysis of the femur compared to the conventional material of Ti (*n* = 0 and *m* = 0) and amount of the SED increased by the *m* and *n* growth (Table 5). These findings are supported by the previous results reported in [4, 46] which showed that the stiff prostheses provoked less SED in the bone than the prostheses with the lower stiffness. Simões et al. [8, 26] also showed that a prosthesis with a tailorable stiffness produced more SED in the bone compared to the prostheses composed of conventional materials of Ti and chrome-cobalt. El-Sheikh [47] and Simões et al. [8, 26] revealed that the developed stress in the prosthesis declines with the reduction in the stiffness of prosthesis which is consistent with results of the present work. The present work demonstrated that more stress was provoked to the bone and cement due to the prosthesis stiffness reduction which was also reported in [4, 8, 26] (Tables 6 and 7). The medial side of the femur carried more stress than the lateral side of the femur which was a similar trend to the findings in [48]. Kuiper and Huiskes [25] and Hedia et al. [27, 28] showed that less interface stress induced to the prosthesis-bone interface by FG prostheses which were in agreement with presented results in this study. Hedia et al. [28] also noted that the longitudinal FG prostheses induced less interface stress at the prosthesis-bone interface that is in agreement with the presented findings (Table 8).

The present study had encountered numerous difficulties in modeling the femur implanted with FG femoral prostheses and presenting the results pertaining to the longitudinal, radial, and longitudinal-radial FG prostheses. However, certain limitations remained, such as material properties and load simplification, as well as static analysis and study of single prosthesis. These simplifications were also found elsewhere [3, 33, 35, 42] and exploited to save time in the modeling process. The loads simplification has been validated against in vivo data by Heller et al. [49] and showed an error of less than 10%.

## 5. Conclusion

The FG prostheses provoked more SED in the bone and showed a better performance that preserves femur from resorption by volume fraction gradient exponent growth. The developed stress in the femoral stem was declined by the volume fraction gradient exponent growth. On other hand, more stress was stimulated to the bone and cement layer with volume fraction gradient exponent increase. The induced interface stresses decreased at the prosthesis-bone interface by volume fraction gradient exponent growth in the noncemented prostheses fixation method, while they showed a limited change in the cemented prostheses fixation methods. However, more interface stress was developed to the cement-bone interface than the prosthesis-cement interface in the cemented prostheses fixation methods. The radial volume fraction gradient exponent was more influential than the longitudinal one. The medial side of the prostheses and bone carried more stress and stair climbing was more harmful compared to the normal walking.

## Figures and Tables

**Table 1:** Some features of the finite element mesh.

Material	Fixation method	Approximate global size (mm)	Maximum deviation factor	Minimum size factor	Number of elements
Cement	—	2.0	0.02	0.1	107556
Femoral prosthesis	—	1.5	0.05	0.1	631470
Femur	Cemented	2.0	0.05	0.1	761218
	Noncemented	2.0	0.05	0.1	792849
Total number of elements	Cemented	—	—	—	1500244
	Noncemented	—	—	—	1424319

**Table 2:** Normal walking-frame of maximum contact force [32, 33].

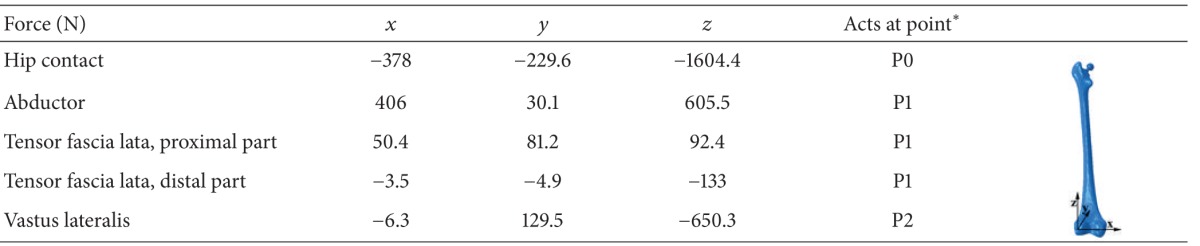

∗Presented in Figure 2.

**Table 3:** Stairs climbing-frame of maximum torsional moment [32, 33].

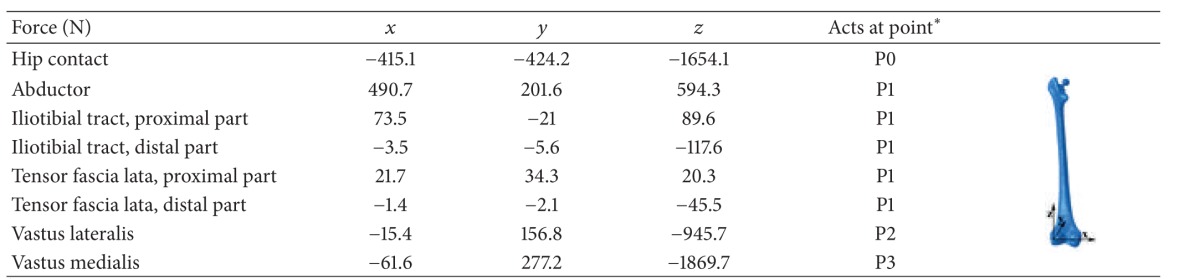

∗Presented in Figure 1.

**Table 4:** Material properties of implanted femur components.

Material	Plane	Modulus of elasticity (*E*) [GPa]	Modulus of rigidity (*G*) [GPa]	Poisson's ratio (*v*)	Ref.
Cortical bone	*xx*	11.5	3.60	0.51	[34, 35]
	*yy*	11.5	3.30	0.31	
	*zz*	17.0	3.30	0.31	
Spongy bone	—	2.13	—	0.30	
Cement	—	2.70	—	0.35	
Titanium alloy (Ti)	—	110	—	0.30	
Hydroxyapatite (HA)	—	10	—	0.30	[30]

**Table 5:** Summary of von Mises stress on the femoral stem.

	m*	Lateral				Medial			
		n*							
		0.0	0.1	0.5	1.0	0.0	0.1	0.5	1.0
Cemented									
Normal waking	0.0	48.9	39.9	18.6	9.6	53.2	43.7	21.9	12.6
	0.1	46.7	38.7	18.2	9.5	49.4	43.5	22.0	12.5
	0.5	42.6	35.0	16.6	9.3	46.5	39.9	21.0	12.3
	1.0	39.1	33.5	15.8	9.2	43.6	37.4	20.0	12.1
Stair climbing	0.0	51.0	42.8	21.5	12.4	76.7	36.5	29.9	16.4
	0.1	48.2	41.6	21.6	12.3	75.4	35.1	16.3	16.0
	0.5	45.3	39.1	20.6	11.9	69.7	32.1	56.9	13.7
	1.0	42.1	38.2	19.5	11.6	62.4	33.7	15.6	13.7
Noncemented									
Normal waking	0.0	47.0	38.4	18.2	20.6	61.1	51.6	27.5	17.6
	0.1	45.9	37.7	18.3	17.5	58.6	50.4	27.1	17.9
	0.5	42.2	35.1	17.3	16.0	55.3	47.9	26.0	17.1
	1.0	38.6	32.1	16.2	16.1	52.3	45.1	24.9	16.8
Stair climbing	0.0	44.2	36.1	17.0	14.8	60.6	51.1	27.1	17.0
	0.1	43.8	35.3	17.8	14.7	58.5	50.3	26.8	18.5
	0.5	39.9	32.7	16.0	14.8	54.6	47.6	25.7	16.3
	1.0	36.4	29.8	15.2	14.8	51.6	44.6	24.5	16.1

*n: radial volume fraction gradient exponent.

*m: longitudinal volume fraction gradient exponent.

**Table 6:** Summary of maximum and minimum principal stress on the femur.

		m*	Maximum principal stress				Minimum principal stress			
			Lateral				Medial			
			n*							
			0.0	0.1	0.5	1.0	0.0	0.1	0.5	1.0

Cemented	Normal waking	0.0	16.4	16.8	18.0	18.9	23.4	24.5	24.4	24.6
		0.1	16.6	17.0	18.2	19.1	23.4	24.7	25.0	25.2
		0.5	17.2	17.5	18.7	19.5	23.6	25.0	25.2	25.4
		1.0	17.9	18.8	19.3	19.9	23.9	25.0	25.5	25.6
	Stair climbing	0.0	19.5	19.9	21.2	22.1	46.7	46.7	46.5	46.7
		0.1	19.7	20.1	21.3	22.2	46.4	47.0	47.4	47.7
		0.5	20.2	20.6	21.7	22.6	46.7	47.5	47.8	48.0
		1.0	20.8	21.3	22.2	23.0	47.0	67.5	48.1	48.2

Cementless	Normal waking	0.0	10.2	10.3	10.6	10.8	9.8	10.0	10.8	13.6
		0.1	10.4	10.4	10.5	10.6	10.9	13.9	14.5	14.9
		0.5	11.0	10.5	10.5	10.6	11.0	15.5	15.5	15.3
		1.0	10.9	10.6	10.7	10.7	11.4	15.9	15.5	15.4
	Stair climbing	0.0	10.0	10.1	10.6	10.9	25.0	25.0	25.1	25.1
		0.1	10.5	10.6	10.8	11.0	24.1	24.5	24.7	24.7
		0.5	11.5	10.9	11.0	11.0	23.8	24.1	24.4	24.6
		1.0	11.2	10.9	10.9	10.9	23.3	24.0	24.3	24.7

**Table 7:** Summary of maximum principal stress on the cement layer.

	m*	Internal				External			
		n*							
		0.0	0.1	0.5	1.0	0.0	0.1	0.5	1.0
Normal waking	0.0	4.5	4.6	4.8	5.0	3.6	3.7	3.8	4.0
	0.1	4.8	4.9	5.0	5.2	3.7	3.7	3.9	4.0
	0.5	4.9	5.0	5.2	5.3	3.7	3.8	3.9	4.1
	1.0	5.0	5.1	5.3	5.3	3.9	3.9	4.0	4.1

Stair climbing	0.0	3.4	3.5	3.6	3.7	4.7	4.8	4.9	5.1
	0.1	3.5	3.5	3.6	3.7	4.7	4.8	5.0	5.1
	0.5	3.5	3.5	3.7	3.8	4.9	4.9	5.0	5.2
	1.0	3.6	3.6	3.7	3.8	5.0	5.1	5.1	5.2

*n: radial volume fraction gradient exponent.

*m: longitudinal volume fraction gradient exponent.

**Table 8:** Summary of shear stresses at the prosthesis-cement and cement-bone interfaces.

	Fixation	Cemented								Noncemented			
	m*	Prosthesis-cement				Cement-bone				Bone-prosthesis			
		n*											
		0	0.1	0.5	1.0	0.0	0.1	0.5	1.0	0	0.1	0.5	1.0
Normal waking	0.0	1.5	1.5	1.6	1.6	1.6	1.7	1.7	1.7	8.3	8.1	7.0	6.4
	0.1	1.5	1.5	1.6	1.6	1.6	1.7	1.7	1.7	7.1	7.1	6.1	5.6
	0.5	1.5	1.5	1.6	1.6	1.7	1.7	1.7	1.7	6.5	5.9	5.1	4.6
	1.0	1.6	1.6	1.6	1.6	1.7	1.6	1.7	1.7	5.4	4.5	4.0	3.7

Stair climbing	0.0	1.7	1.7	1.7	1.7	2.2	2.3	2.3	2.3	10.9	10.9	10.1	9.2
	0.1	1.7	1.7	1.7	1.7	2.3	2.3	2.3	2.3	9.9	9.6	9.0	8.7
	0.5	1.7	1.7	1.7	1.7	2.3	2.2	2.3	2.3	9.5	9.2	8.0	7.0
	1.0	1.7	1.7	1.7	1.7	2.3	2.3	2.3	2.3	7.3	6.8	5.6	3.6

*n: radial volume fraction gradient exponent.

*m: longitudinal volume fraction gradient exponent.
